# Supplementary material for: Jamestown Canyon virus is transmissible by Aedes aegypti and is only moderately blocked by Wolbachia co-infection
Source: PLoS Negl Trop Dis. 2023 Sep 5;17(9):e0011616. doi: 10.1371/journal.pntd.0011616 (PMC10503764; doi:10.1371/journal.pntd.0011616)
Supplement: S1 Table — W- = wildtype line, W+ = Wolbachia infected. Use of mosquitoes for different experiments is listed. (DOCX) [file pntd.0011616.s001.docx]

| lines | rep | N | Averaged viral load (copies/mosquito) | Standard deviation | Experiments |
| --- | --- | --- | --- | --- | --- |
| *W*- | 1 | 4 | 3421751 | 1425887 | 1^st^ replicate of viral quantification |
| *W*+ | 1 | 4 | 3878587 | 1732395 | 1^st^ replicate of viral quantification |
| *W*- | 2 | 4 | 3488288 | 2648234 | 2^nd^ replicate of viral quantification, immune gene expression, and *Wolbachia* density |
| *W*+ | 2 | 4 | 3091115 | 1707324 | 2^nd^ replicate of viral quantification, immune gene expression, and *Wolbachia* density |
| *W*- | 3 | 4 | 4844385 | 2059773 | Longevity |
| *W*+ | 3 | 4 | 6545050 | 1946221 | Longevity |
