# Supplementary material for: Jamestown Canyon virus is transmissible by Aedes aegypti and is only moderately blocked by Wolbachia co-infection
Source: PLoS Negl Trop Dis. 2023 Sep 5;17(9):e0011616. doi: 10.1371/journal.pntd.0011616 (PMC10503764; doi:10.1371/journal.pntd.0011616)
Supplement: S2 Table — rps17 is the reference gene. (DOCX) [file pntd.0011616.s002.docx]

| Primer name | Primer sequence forward (5’-3’) | Primer sequence reverse (5’-3’) | Product length | Efficiency | Reference |
| --- | --- | --- | --- | --- | --- |
| MYD88 | GGAGCTTCCTGCAAACCTAA | TATGGCATCTTCCAGCTTGTC | 94 | 102.4% | Alto et al [1] |
| IMD | TGGTCAACCTGTTATGGCAA | GGGTTGACTTTGTCGTCGTT | 86 | 117.1% | Cabral et al [2] |
| hop | CCGGACTTTATCGAGCTGTC | ATCTGGTTCACTCCGTCGTC | 113 | 117.2% | Jupatanakul et al [3] |
| AGO2 | GGCTGCTCACCCAATGTATCAAGA | AACCGTTCGTTTTGGCGTTGAT | 105 | 91.5% | McFarlane et al [4] |
| Vago | GCTAACTTTGATTACCAACCTGGG | TTTGTGCTGCTTGATCGCAG | 84 | 124.2% | Newly designed |
| Rps17 | TCCGTGGTATCTCCATCAAGCT | CACTTCCGGCACGTAGTTGTC | 68 | 96.6% | Terradas et al [5] |

1. Alto BW, Civana A, Wiggins K, Eastmond B, Shin D. Effect of oral infection of Mayaro virus on fitness correlates and expression of immune-related genes in *Aedes aegypti*. Viruses. 2020;12: 719. doi:10.3390/v12070719

2. Cabral S, De Paula A, Samuels R, Da Fonseca R, Gomes S, Silva JR, et al. *Aedes aegypti* (Diptera:

Culicidae) Immune responses with different feeding regimes following infection by the

entomopathogenic fungus *Metarhizium anisopliae*. Insects. 2020;11: 95. doi:10.3390/insects11020095

3. Jupatanakul N, Sim S, Angleró-Rodríguez YI, Souza-Neto J, Das S, Poti KE, et al. Engineered *Aedes*

*aegypti* JAK/STAT pathway-mediated immunity to dengue virus. PLOS Negl Trop Dis. 2017;11:

e10005187.

doi:10.1371/JOURNAL.PNTD.0005187

4. McFarlane M, Arias-Goeta C, Martin E, O’Hara Z, Lulla A, Mousson L, et al. Characterization of *Aedes*

*aegypti* innate-immune pathways that limit Chikungunya virus replication. PLOS Negl Trop Dis. 2014;8:

e2994. doi:10.1371/journal.pntd.0002994

5. Terradas G, Joubert DA, McGraw EA. The RNAi pathway plays a small part in *Wolbachia*-mediated

blocking of dengue virus in mosquito cells. Sci Rep. 2017;7: 43847.

doi:10.1038/srep43847
