# Supplementary material for: Jamestown Canyon virus is transmissible by Aedes aegypti and is only moderately blocked by Wolbachia co-infection
Source: PLoS Negl Trop Dis. 2023 Sep 5;17(9):e0011616. doi: 10.1371/journal.pntd.0011616 (PMC10503764; doi:10.1371/journal.pntd.0011616)
Supplement: S3 Table — (DOCX) [file pntd.0011616.s003.docx]

| Virus load comparisons for the first replicate | | | | | | |
| --- | --- | --- | --- | --- | --- | --- |
| tissue | dpi | viral load | | prevalence | | |
|  |  | Mann-Whitney-U-Test | | Chi-squared test | | |
|  |  | Z | p | 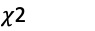 | df | p |
| Abdomen | 3 | 1.98 | 0.048 | 0.52 | 1 | 0.47 |
|  | 7 | -0.88 | 0.38 | 1.30 | 1 | 0.26 |
|  | 10 | 2.58 | 0.01 | 0.00 | 1 | 1 |
|  | 14 | 2.73 | 0.006 | 6.62 | 1 | 0.01 |
| Head & Thorax | 3 | 2.12 | 0.034 | 14.40 | 1 | < 0.001 |
|  | 7 | 1.57 | 0.017 | --- | 1 | --- |
|  | 10 | -0.45 | 0.66 | 1.40 | 1 | 0.24 |
|  | 14 | 5.58 | <0.001 | 3.49 | 1 | 0.062 |
| Saliva | 3 | 0.37 | 0.72 | 0.45 | 1 | 0.50 |
|  | 7 | -0.97 | 0.33 | 13.20 | 1 | <0.001 |
|  | 10 | 1.39 | 0.16 | 0.27 | 1 | 0.60 |
|  | 14 | -2.82 | 0.005 | 1.67 | 1 | 0.20 |
| Legs | 3 | -2.52 | 0.012 | --- | 1 | -- |
|  | 7 | -2.43 | 0.015 | 0 | 1 | 1 |
|  | 10 | 0.77 | 0.44 | 0 | 1 | 1 |
|  | 14 | 0.75 | 0.46 | 0 | 1 | 1 |
| Viral load comparisons for the second replicate | | | | | | |
| tissue | dpi | viral load | | prevalence | | |
|  |  | Mann-Whitney-U-Test | | Chi-squared test | | |
|  |  | Z | p | 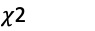 | df | p |
| Abdomen | 3 | 4.15 | <0.001 | 17.80 | 1 | <0.001 |
|  | 7 | -0.96 | 0.34 | 0 | 1 | 1 |
|  | 10 | 1.89 | 0.058 | --- | 1 | --- |
|  | 14 | 2.06 | 0.039 | 1.40 | 1 | 0.24 |
| Head & Thorax | 3 | 4.10 | <0.001 | 12.80 | 1 | <0.001 |
|  | 7 | -0.97 | 0.33 | 0 | 1 | 1 |
|  | 10 | 0.19 | 0.85 | --- | 1 | --- |
|  | 14 | 0.93 | 0.36 | 0 | 1 | 1 |
