# Supplementary material for: Jamestown Canyon virus is transmissible by Aedes aegypti and is only moderately blocked by Wolbachia co-infection
Source: PLoS Negl Trop Dis. 2023 Sep 5;17(9):e0011616. doi: 10.1371/journal.pntd.0011616 (PMC10503764; doi:10.1371/journal.pntd.0011616)
Supplement: S4 Table — (DOCX) [file pntd.0011616.s004.docx]

| Gene expression comparisons | | | |
| --- | --- | --- | --- |
| Gene name (ID) | dpi | Tukey's HSD test | |
|  |  | t | p |
| *MYD88* (AAEL007768) | 3 | 1.29 | 0.20 |
|  | 7 | 0.93 | 0.36 |
|  | 10 | -1.91 | 0.057 |
|  | 14 | 1.87 | 0.063 |
| *IMD*  (AAEL010083) | 3 | 1.07 | 0.29 |
|  | 7 | 1.71 | 0.089 |
|  | 10 | -2.09 | 0.038 |
|  | 14 | 1.87 | 0.063 |
| *hopscotch* (AAEL012553) | 3 | 2.77 | 0.006 |
|  | 7 | 0.64 | 0.52 |
|  | 10 | 2.11 | 0.036 |
|  | 14 | 5.06 | <0.001 |
| *AGO2*  (AAEL017251) | 3 | 1.67 | 0.096 |
|  | 7 | -1.82 | 0.069 |
|  | 10 | 0.77 | 0.44 |
|  | 14 | 3.89 | <0.001 |
| *AeVago1* (AAEL000200) | 3 | 2.38 | 0.018 |
|  | 7 | 0.046 | 0.96 |
|  | 10 | 1.91 | 0.058 |
|  | 14 | 3.72 | <0.001 |
