# Supplementary material for: Jamestown Canyon virus is transmissible by Aedes aegypti and is only moderately blocked by Wolbachia co-infection
Source: PLoS Negl Trop Dis. 2023 Sep 5;17(9):e0011616. doi: 10.1371/journal.pntd.0011616 (PMC10503764; doi:10.1371/journal.pntd.0011616)
Supplement: S1 Text — (DOCX) [file pntd.0011616.s005.docx]

We used a total reaction volume of 10 µL, consisting of 5μL of 2x PerfeCTa SYBR Green SuperMix (Quantabio), 0.2 µL (1µM) of each forward and reverse primer, 0.2µL of RT enzyme and 2.4 µL of DNAse RNAse-free ultrapure water, and 2 µL of 20ng/µL RNA template. Samples were analyzed through absolute quantification, by comparison to serial dilutions of a JCV segment S gene product, ranging from 10^8^ to 10^1^ copies, generated as previously described. Standards were aliquoted and frozen for one-time use. All reactions were carried out in duplicate.

The thermocycling conditions consisted of an initial cDNA synthesis step at 50 ˚C for 5 minutes (Ramp Rate: 4.4 ˚C/s), followed by a Taq inactivation at 95 ˚C for 2 minutes (Ramp Rate: 4.4 ˚C/s) and 40 amplification cycles of 95 ˚C for 3 seconds (Ramp Rate: 4.4 ˚C/s), and 60 ˚C for 30 seconds (Ramp Rate: 2.2 ˚C/s). Melting curve analysis consisted of 95 ˚C for 15 seconds (Ramp Rate: 4.4 ˚C/s), 60 ˚C for 15 seconds (Ramp Rate: 2.2 ˚C/s), and 95 ˚C continuous (Ramp Rate: 0.11 ˚C/s).
