# Supplementary figures and images for: Jamestown Canyon virus is transmissible by Aedes aegypti and is only moderately blocked by Wolbachia co-infection
Source: PLoS Negl Trop Dis. 2023 Sep 5;17(9):e0011616. doi: 10.1371/journal.pntd.0011616 (PMC10503764; doi:10.1371/journal.pntd.0011616)

**
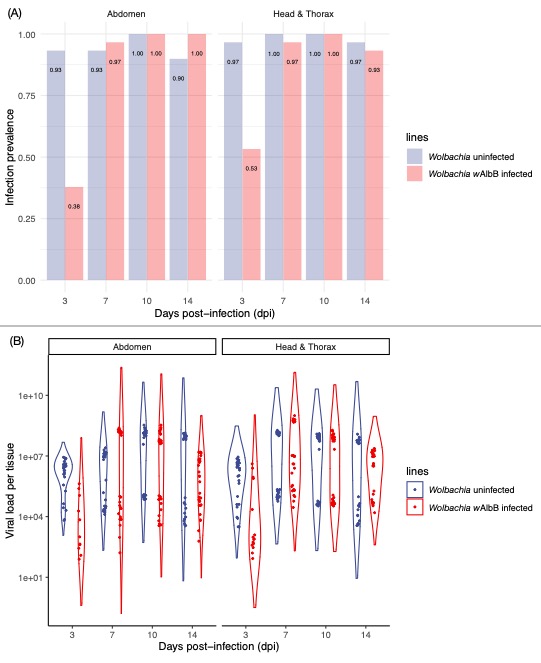
**

Supplement: S1 Fig — (A) Jamestown Canyon virus (JCV) infection prevalence in adult female mosquito saliva and legs at 3, 7, 10, and 14 days post-infection (dpi). n = 30 per treatment. In both abdomen and head & thorax, there were significant effects for both Wolbachia infection (abdomen: z = -2.63, p = 0.008; head & thorax: z = -3.14, p = 0.002) and dpi (abdomen: z = 4.50, p < 0.001; head & thorax: z = 3.66, p < 0.001) on infection prevalence. (B) For viral load, in either abdomen and head & thorax, there was no significant impact of Wolbachia infection (abdomen: t = 0.22, p = 0.83; head & thorax: t = 1.94, p = 0.053) or dpi (abdomen: t = 1.85, p = 0.066; head & thorax: t = - 0.97, p = 0.33) but a significant interaction was found for head & thorax (abdomen: t = 0.22, p = 0.83; head & thorax: t = 2.67, p = 0.008). (DOCX) [file pntd.0011616.s006.docx]

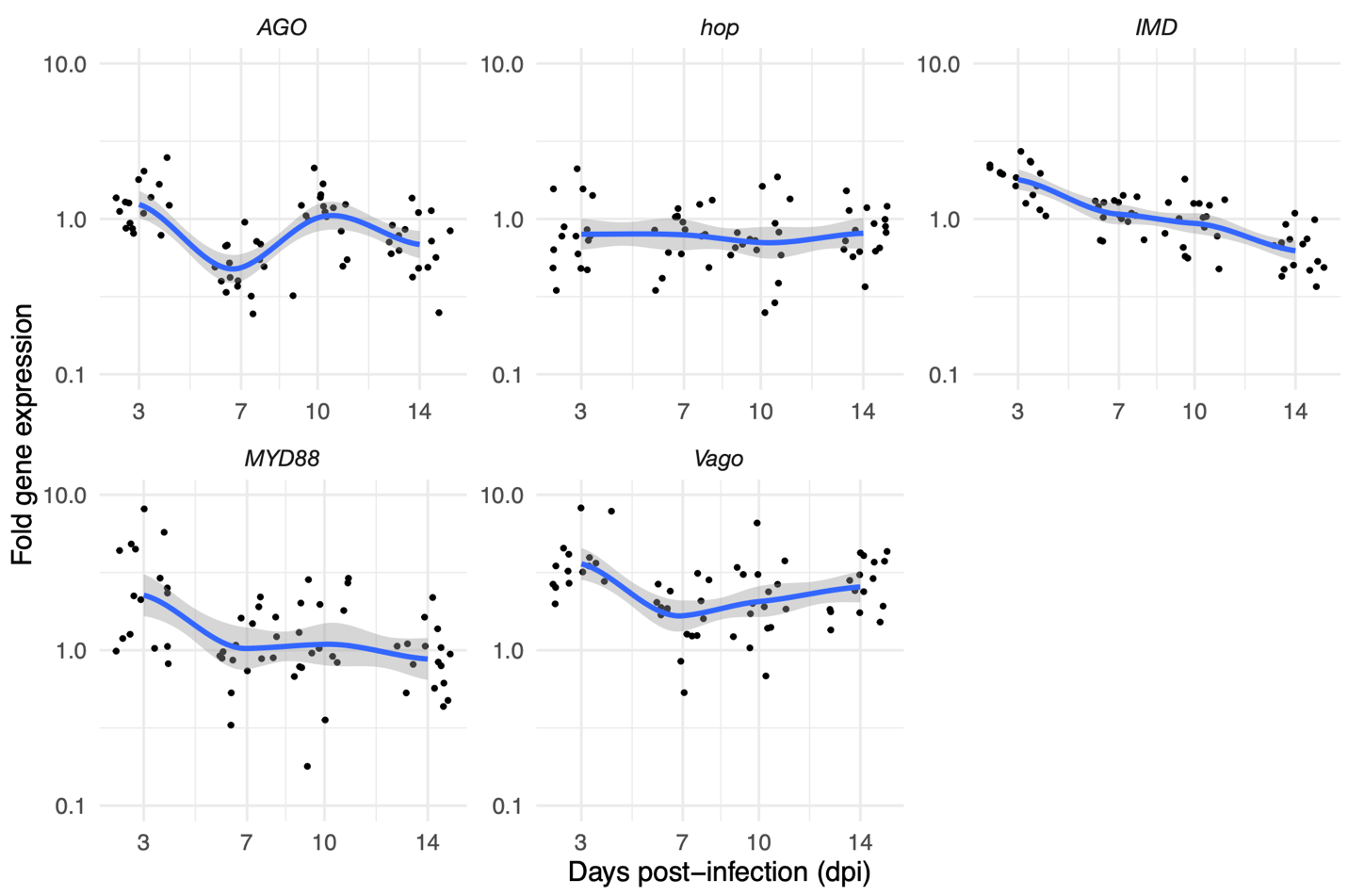

Supplement: S2 Fig — The maximum change of gene expression levels in response to Wolbachia infection was less than 10-fold for all genes at all dpi. n = 16 per treatment. Significant differences were found between Wolbachia infected and uninfected lines (F1,600 = 10.39, p = 0.001), among different genes (F4,600 = 27.00, p < 0.001), and among different dpi (F3,600 = 13.41, p < 0.001). When genes were considered separately, gene AGO (F1,120 = 8.67, p = 0.004), hop (F1,120 = 9.78, p = 0.002), and Vago (F1,120 = 59.47, p < 0.001) were affected by Wolbachia infection, but not for gene MYD88 (F1,120 = 3.81, p = 0.053) and IMD (F1,120 = 0.20, p = 0.66). (DOCX) [file pntd.0011616.s007.docx]

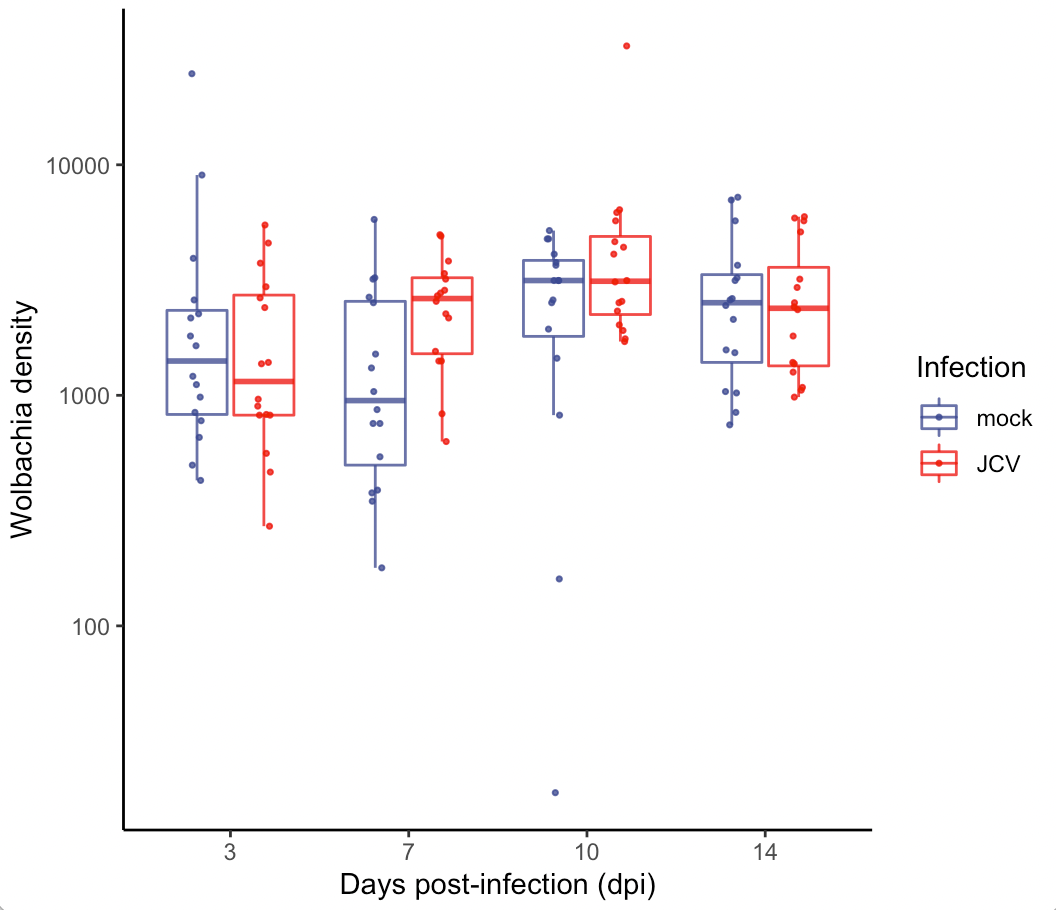

Supplement: S3 Fig — n = 16 per treatment. (DOCX) [file pntd.0011616.s008.docx]
